# Taxonomic studies on the genus *Trilacuna* (Araneae, Oonopidae) from Myanmar

**DOI:** 10.3897/zookeys.960.54053

**Published:** 2020-08-17

**Authors:** Yanfeng Tong, Shuqiang Li, Dongju Bian

**Affiliations:** 1 Life Science College, Shenyang Normal University, Shenyang 110034, China Shenyang Normal University Shenyang China; 2 Southeast Asia Biological Diversity Research Institute, Chinese Academy of Sciences, Yezin, Nay Pyi Taw 05282, Myanmar Southeast Asia Biological Diversity Research Institute, Chinese Academy of Sciences Yezin Myanmar; 3 Institute of Zoology, Chinese Academy of Sciences, Beijing 100101, China Institute of Zoology, Chinese Academy of Sciences Beijing China; 4 CAS Key Laboratory of Forest Ecology and Management, Institute of Applied Ecology, Shenyang 110016, China Institute of Applied Ecology Shenyang China

**Keywords:** goblin spiders, new species, Oonopinae, taxonomy

## Abstract

Six species of the genus *Trilacuna* Tong & Li, 2007 are reported from Myanmar, including four new species: *T. besucheti* Grismado & Piacentini, 2014 (♂♀), *T. changzi* Tong & Li, **sp. nov.** (♂♀), *T. hponkanrazi* Tong & Li, **sp. nov.** (♂♀), *T. loebli* Grismado & Piacentini, 2014 (♀), *T. triseta* Tong & Li, **sp. nov.** (♂), and *T. zhigangi* Tong & Li, **sp. nov.** (♀). Morphological descriptions and photographic illustrations of the new species are given. All types are preserved in the Institute of Zoology, Chinese Academy of Sciences in Beijing (IZCAS).

## Introduction

Of the 1850 spider species of family Oonopidae Simon, 1890 known worldwide (Li 2020), 10 have been previously recorded from Myanmar: *Gamasomorpha inclusa* (Thorell, 1887), *G. psyllodes* Thorell, 1897, *G. sculptilis* Thorell, 1897, *Kachinia mahmolae* Tong & Li, 2018, *K. putao* Tong & Li, 2018, *Opopaea kanpetlet* Tong & Li, 2020, *O. zhigangi* Tong & Li, 2020, *Promolotra hponkanrazi* Tong & Li, 2020, *P. shankhaung* Tong & Li, 2020, and *Xestaspis parmata* Thorell, 1890. The current article investigates species of the genus *Trilacuna* Tong & Li, 2007 that were collected in Myanmar and includes descriptions and illustrations of four new species.

The spider genus *Trilacuna* was established to accommodate two new species from Southwest China (Tong and Li 2007). Subsequently, additional species have been described: seven from Thailand, Malaysia, and Sumatra (Eichenberger and Kranz-Baltensperger 2011), two from Vietnam (Tong and Li 2013), seven from Bhutan, India, Nepal, and Pakistan (Grismado et al. 2014), one from Iran (Malek-Hosseini et al. 2015), one from Korea (Seo 2017), and 10 from Southwest China (Tong et al. 2018, 2019; Liu et al. 2019). Currently, the genus *Trilacuna* comprises 30 species, all of which are known from Asia (WSC 2020).

## Methods

The specimens were examined in 95% ethanol using a Leica M205C stereomicroscope. Details were studied with an Olympus BX51 compound microscope. Photos were taken with a Canon EOS 750D zoom digital camera (18 megapixels) mounted on an Olympus BX51 compound microscope. Vulvae were cleared in lactic acid. Scanning electron microscope images (SEM) were taken in a high vacuum under a Hitachi TM3030 after critical point drying and gold-palladium coating. All measurements were taken using an Olympus BX51 compound microscope and are given in millimeters in the text. The materials are preserved in the Institute of Zoology, Chinese Academy of Sciences in Beijing (**IZCAS**).

The following abbreviations are used in the text and figures:

**ab** anterior branch

**ALE** anterior lateral eyes

**ALE–PLE** distance ALE–PLE

**ap** apodeme

**as** anterior sclerite

**boc** booklung covers

**bts** bent thick setae

**cmp** clypeus median projection

**cos** comb-like setae

**db** dorsal branch

**ds** dorsal setae

**emb** embolus

**esb** elevated seta base

**glo** globular structure

**lb** lateral branch

**ldi** labium deep incision

**ls** long setae

**mb** median branch

**ml** median lobe

**PLE** posterior lateral eyes

**PME** posterior median eyes

**psp** posterior spiracle;

**sep** semicircular plate

**smb** small median branch

**svl** small ventral lobe

**tba** transverse bars

**tp** triangular plate

**tsc** transverse sclerite

**vb** ventral branch

**vs** ventral setae

## Taxonomy

### Family Oonopidae Simon, 1890

#### Genus Trilacuna

Taxon classificationAnimaliaAraneaeOonopidae

Tong & Li, 2007

48BDD660-340F-51B6-800A-65037036428E

Trilacuna Tong & Li, 2007: 333; Grismado et al. 2014: 26.

##### Type species.

*Trilacuna rastrum* Tong & Li, 2007

##### Diagnosis.

*Trilacuna* differs from other oonopid genera, except those of the “*Dysderoides* complex” (including *Bannana* Tong & Li, 2015, *Dysderoides* Fage, 1946, *Himalayana* Grismado, 2014, and *Trilacuna*), by the enlarged male palpal femur, the very complex embolus-conductor system, and the notched labium. Males differ from the other genera of the “*Dysderoides* complex” by usually lacking the furrow connecting the posterior tracheal spiracles, and females differ by having a long postgastric scutum covering almost the whole ventral abdomen (Grismado et al. 2014; Tong et al. 2019).

##### Composition.

34 species, including four described here.

##### Distribution.

Iran to the Korean Peninsula.

#### Trilacuna besucheti

Taxon classificationAnimaliaAraneaeOonopidae

Grismado & Piacentini, 2014

8F92AB36-4B65-56BE-8005-843183B4C1FF

**Figure 1.**
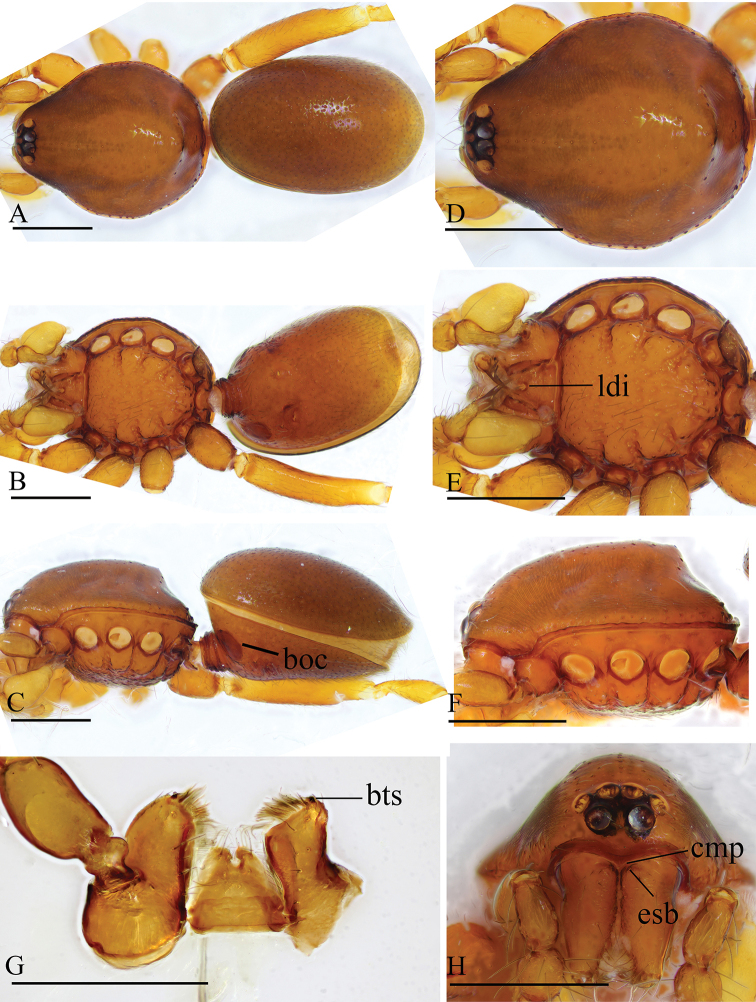
*Trilacuna besucheti*, male (IZCAS AR-25151) **A–C** habitus in dorsal, ventral, and lateral views **D–F, H** prosoma in dorsal, ventral, lateral, and anterior views **G** labium and endites in ventral view. Abbreviations: boc = booklung covers; bts = bent thick setae; cmp = clypeus median projection; esb = elevated seta base; ldi = labium deep incision. Scale bars: 0.4 mm (**A–F, H**); 0.2 mm (**G**).

**Figure 2.**
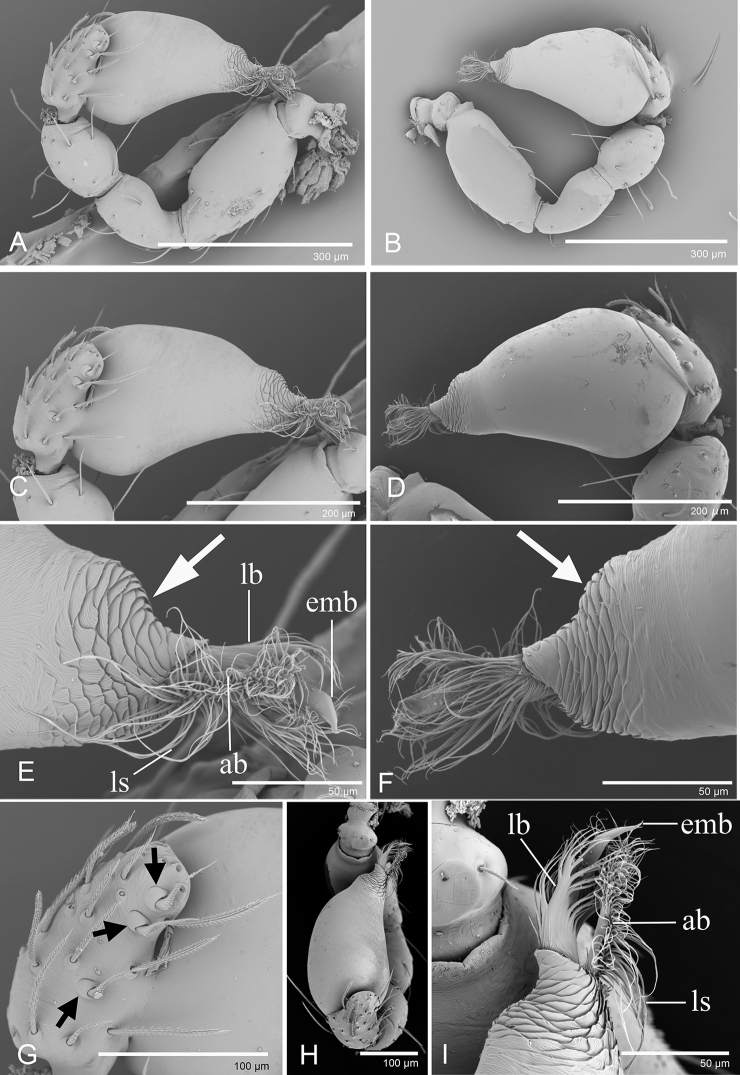
*Trilacuna besucheti*, left male palp, SEM (IZCAS AR-25151) **A, B, H** prolateral, retrolateral, and dorsal views **C, D** palpal bulb in prolateral and retrolateral views **E, F, I** distal part of palpal bulb in prolateral, retrolateral, and dorsal views (white arrows show the circular scale-like structure) **G** cymbium in prolateral view (black arrows show the large setae bases). Abbreviations: ab = anterior branch; emb = embolus; lb = lateral branch; ls = long setae.

**Figure 3.**
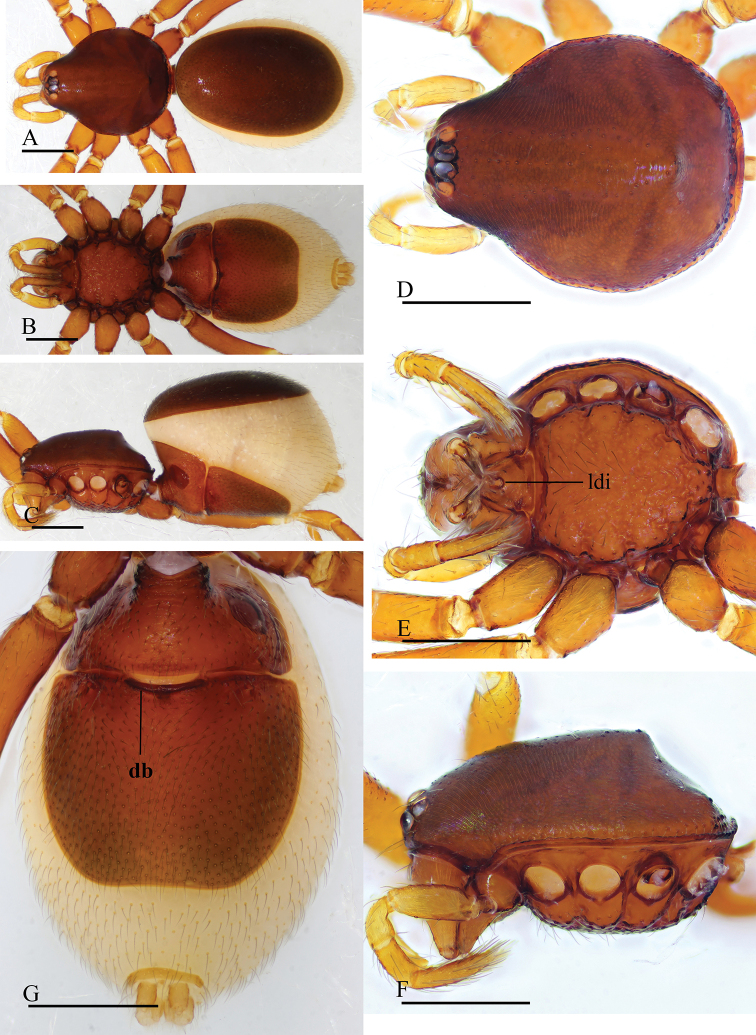
*Trilacuna besucheti*, female (IZCAS AR-25154) **A–C** habitus in dorsal, ventral, and lateral views **D–F** prosoma in dorsal, ventral, and lateral views **G** abdomen in ventral view. Abbreviation: db = darkened band; ldi = labium deep incision. Scale bars: 0.4 mm.

Figs 1 , 2 , 3 , 14A–C , 15A, B , 16E, F

Trilacuna besucheti Grismado & Piacentini, in Grismado et al. 2014: 40, fig. 32A–H, 33A–F, 34A–F, 39C–D

##### Material examined.

3♂1♀, Myanmar, near 1.5 km from the roadside between Kanpetlet and Nat Ma Taung National Park; 21°13.058'N, 93°59.033'E; elevation ca 2420 m; 1.V.2017; Wu J. and Chen Z. leg. (IZCAS AR-25151-25152-25153-25154).

##### Diagnosis.

Males of this species can be recognized by the circular, scale-like structure on the distal part of the bulb (white arrows in Fig. 2E, F) and the cymbium, which has two or three stout, dark setae with large bases (black arrows in Fig. 2G). Females are distinguished by having a darkened band (db) on the posterior margin of the epigastric furrow (Figs 3G, 15A).

##### Description.

See Grismado et al. (2014).

##### Variation.

The specimens from Myanmar have unbranched endites (Fig. 16E, F) and a strongly striated carapace (Figs 1D, F, 3D, F), whereas the specimens from India have distinctly branched endites and a smooth carapace (Grismado et al. 2014: figs 32–34).

##### Distribution.

India (Meghalaya), Myanmar.

#### Trilacuna changzi

Taxon classificationAnimaliaAraneaeOonopidae

Tong & Li sp. nov.

7010CDEC-AD21-5F88-9B5D-FF0C5EA2EA6E

http://zoobank.org/8557BE3E-8492-4401-B95E-C4C9D177DFA6

**Figure 4.**
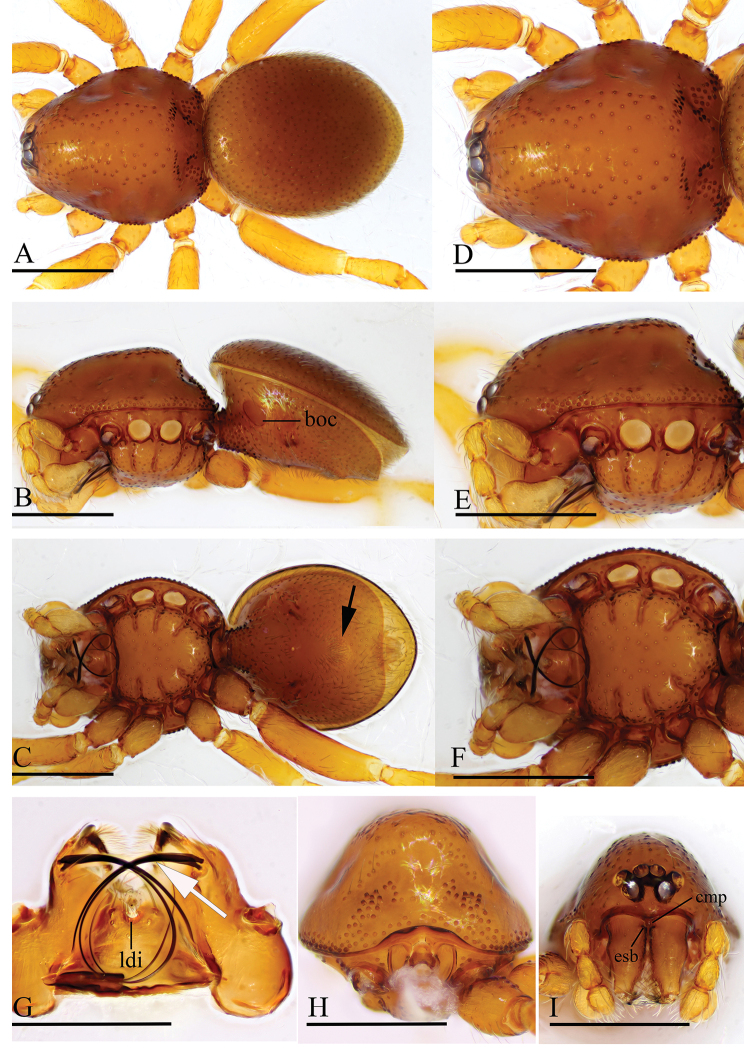
*Trilacuna changzi* sp. nov., male holotype **A–C** habitus in dorsal, lateral, and ventral views; black arrow shows the cluster of densely, short setae **D–F, H, I** prosoma in dorsal, lateral, ventral, posterior, and anterior views **G** labium and endites in ventral view; white arrow shows the long, strongly curved spines. Abbreviations: boc = booklung covers; cmp = clypeus median projection; esb = elevated seta base; ldi = labium deep incision. Scale bars: 0.4 mm (**A–F, H, I**); 0.2 mm (**G**).

**Figure 5.**
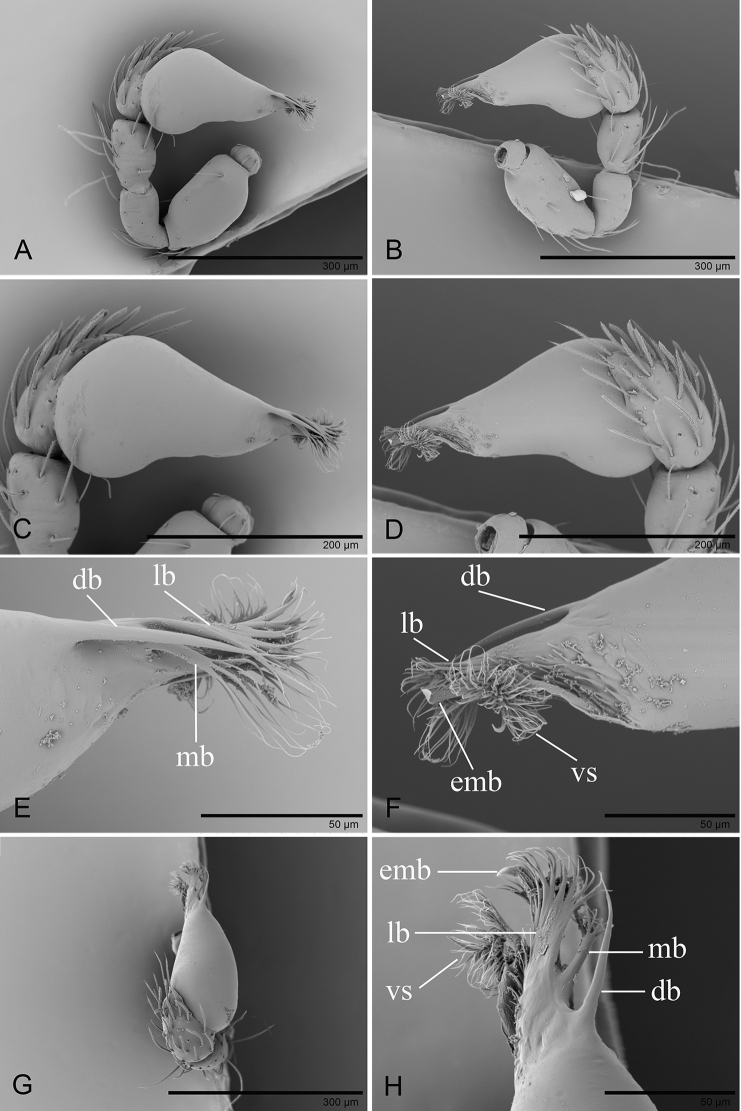
*Trilacuna changzi* sp. nov., male holotype, left palp **A, B, G** prolateral, retrolateral, and dorsal views **C, D** palpal bulb in prolateral and retrolateral views **E, F, H** distal part of palpal bulb in prolateral, retrolateral and dorsal views. Abbreviations: db = dorsal branch; emb = embolus; lb = lateral branch; mb = median branch; vs = ventral setae.

**Figure 6.**
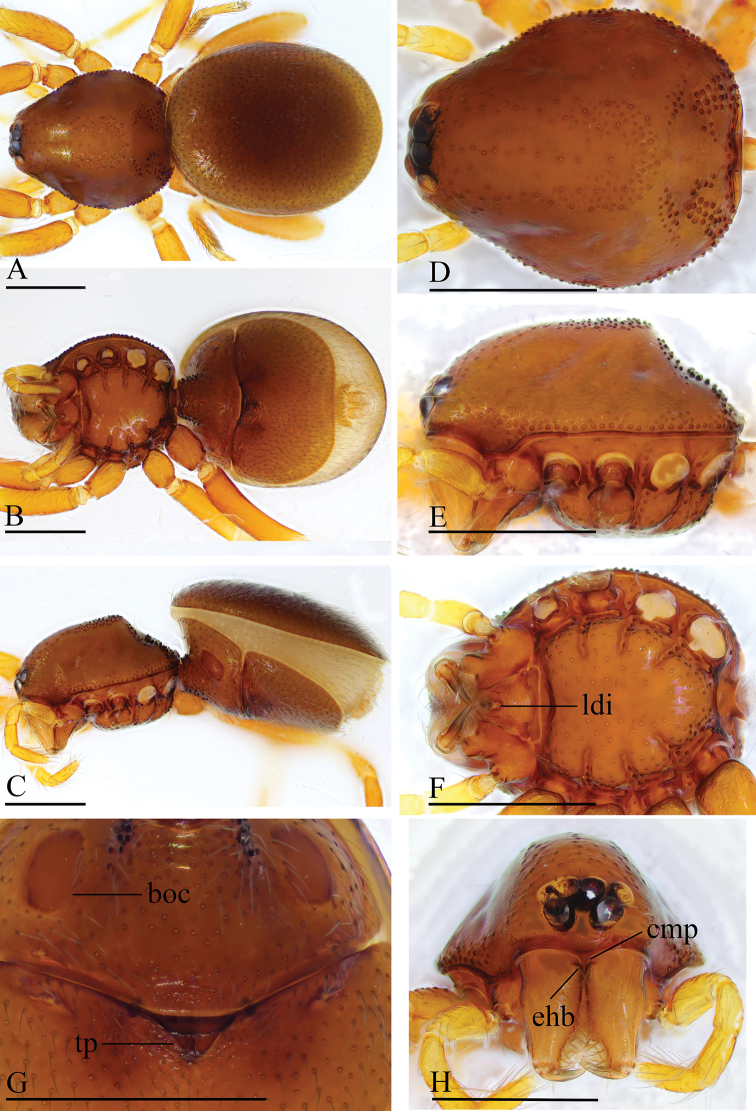
*Trilacuna changzi* sp. nov., female paratype **A–C** habitus in dorsal, ventral, and lateral views **D–F, H** prosoma in dorsal, lateral, ventral, and anterior views **G** abdomen in ventral view. Abbreviations: boc = booklung covers; cmp = clypeus median projection; esb = elevated seta base; ldi = labium deep incision; tp = triangular plate. Scale bars: 0.4 mm.

Figs 4 , 5 , 6 , 14D–F , 15C, D , 16A, B

##### Type material.

***Holotype*** ♂: Myanmar, near 1.5 km from the roadside between Kanpetlet and Nat Ma Taung National Park; 21°13.058'N, 93°59.033'E; elevation ca 2420 m; 1.V.2017; Wu J. and Chen Z. leg. (IZCAS AR-25139). ***Paratype*** 1♀: Myanmar, same data as for holotype (IZCAS AR-25140).

##### Diagnosis.

The new species is similar to *T. mahanadi* Grismado & Piacentini, 2014 but can be distinguished by the long, strongly curved spines on the male endites (Figs 4G, 16A, B), the bare dorsal branch (db) of the embolus system (Figs 5E, F, H, 14E), and the triangular plate (tp) of the female epigastric area (Fig. 6G). The male of *T. mahanadi* has unmodified endites and lacks the dorsal branch of the embolus system, and the triangular plate is lacking in the epigastric area of the female (Grismado et al. 2014: figs 36–38).

##### Description.

**Male. *Body***: yellow-brown, chelicerae and sternum lighter, legs yellow; habitus as in Figure 4A–C; body length 1.56. ***Carapace***: 0.76 long, 0.64 wide; sides smooth, lateral margin rebordered (Fig. 4D); posterior surface with several large setal bases (Fig. 4H). ***Eyes***: ALE largest; PLE and PME nearly equal in size; ALE–PLE separated by less than ALE radius; PME touching each other; posterior eye row recurved as viewed from above, procurved as viewed from front (Fig. 4D, I). ***Clypeus***: height about 0.7 times of ALE diameter, with a triangular, pointed median projection (cmp). ***Mouthparts*** (Figs 4G–I, 16A, B): endites slender, with two long, strongly curved spines. ***Sternum***: (Fig. 4F). ***Abdomen***: 0.82 long, 0.66 wide; booklung covers ovoid, surface smooth (Fig. 4I); dorsal scutum not fused to epigastric scutum; apodemes present, posterior spiracles not connected by groove; epigastric region with a cluster of dense, short setae (Fig. 4C). ***Palp*** (Figs 5, 14D–F): orange; 0.48 long (0.15, 0.11, 0.09, 0.13); femur greatly elongated (width/length = 0.65) (Fig. 5A, B); bulb pear-shaped, tapering apically; embolus system (Fig. 5E, F, H) with a bare dorsal branch (db) in prolateral view, and a small median branch (mb) and laterally curved branch (lb) in dorsal view.

**Female.** Same as male except as noted. ***Habitus***: as in Figure 6A–C; slightly larger than male. ***Body***: length 1.76. ***Carapace***: 0.79 long, 0.67 wide. ***Abdomen***: 1.12 long, 0.82 wide. ***Endites***: unmodified. ***Epigastric area*** (Figs 6G, 15C): with a large, triangular plate (tp). ***Endogyne*** (Fig. 15D): with narrow, transverse sclerite (tsc), an anterior stick-shaped sclerite (as), and a posterior tortuous, tube-like globular structure (glo); transverse bars (tba) with two lateral apodemes (ap).

##### Etymology.

The specific name is derived from Chinese pinyin, “changzi”, which means “long moustache”, referring to the long, curved spines on the male’s endites; noun in apposition.

##### Distribution.

Known only from the type locality.

#### Trilacuna hponkanrazi

Taxon classificationAnimaliaAraneaeOonopidae

Tong & Li sp. nov.

0A187242-ED45-594C-8694-50147D8E8886

http://zoobank.org/13FB4E13-509E-4C85-A8EB-AA4DCD6607BB

**Figure 7.**
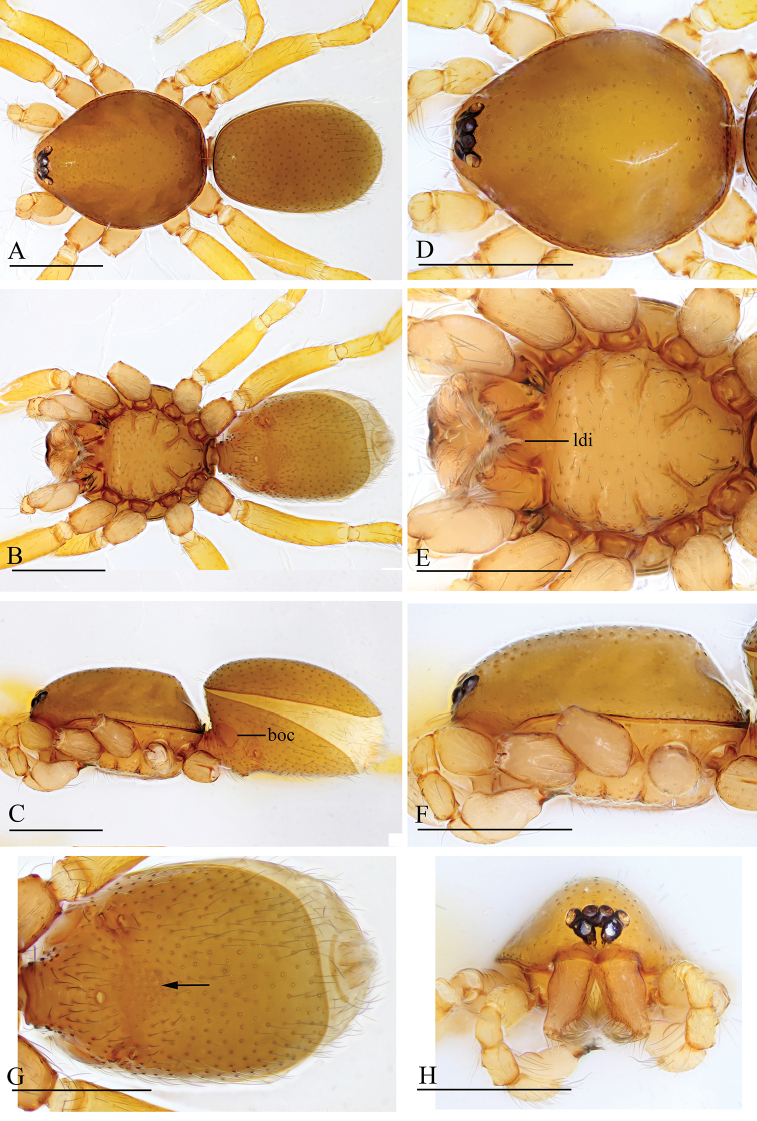
*Trilacuna hponkanrazi* sp. nov., male holotype **A–C** habitus in dorsal, ventral, and lateral views **D–F, H** prosoma in dorsal, ventral, lateral, and anterior views **G** abdomen in ventral view (arrow shows the patches). Abbreviations: boc = booklung covers; ldi = labium deep incision. Scale bars: 0.4 mm.

**Figure 8.**
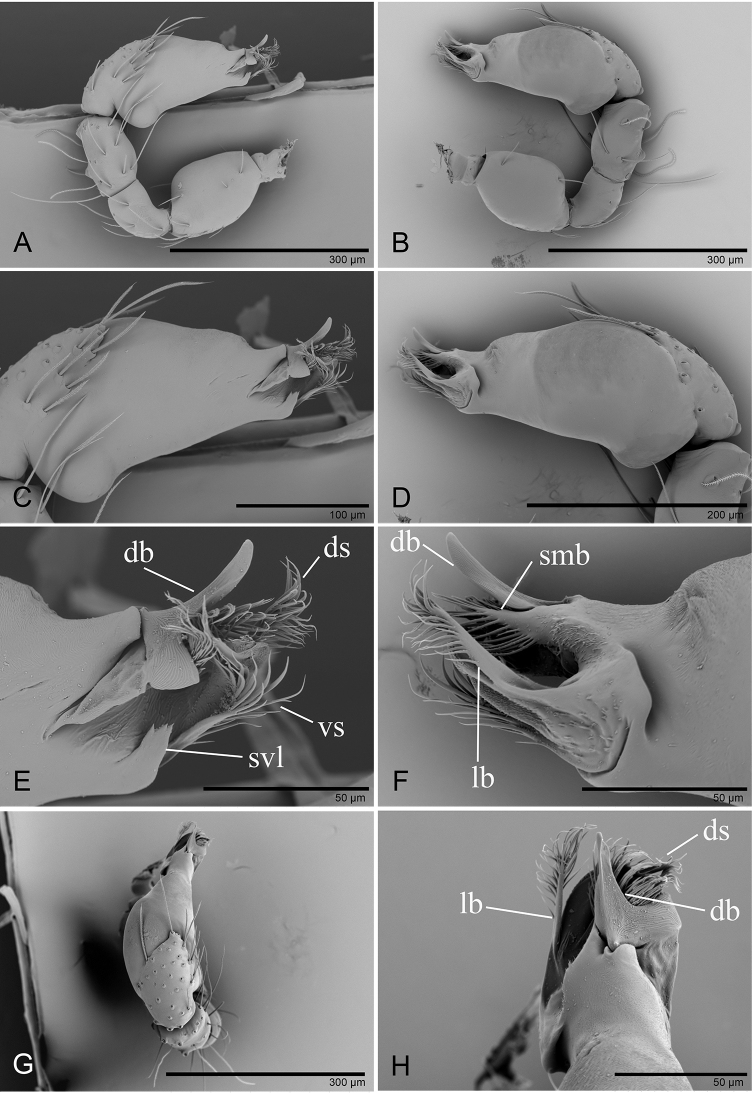
*Trilacuna hponkanrazi* sp. nov., male holotype, left palp, SEM**A, B, G** prolateral, retrolateral and dorsal views **C, D** palpal bulb in prolateral and retrolateral views **E, F, H** distal part of palpal bulb in prolateral, retrolateral, and dorsal views. Abbreviations: db = dorsal branch; ds = dorsal setae; lb= lateral branch; smb= small median branch; svl = small ventral lobe; vs = ventral setae.

**Figure 9.**
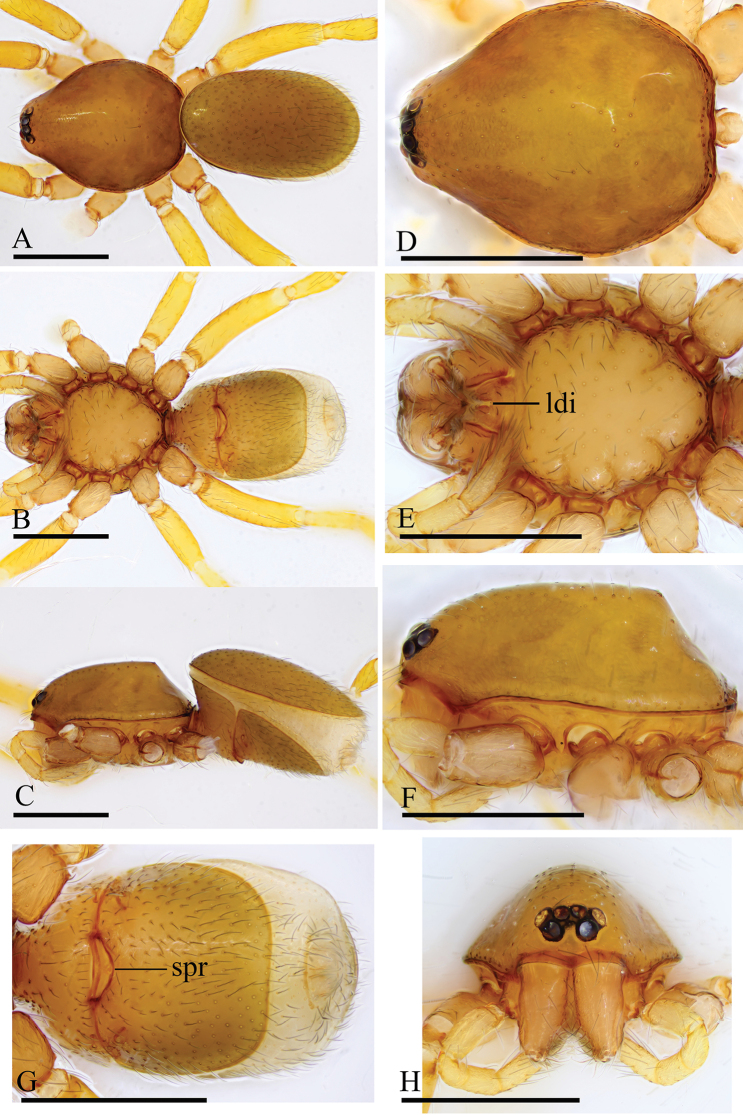
*Trilacuna hponkanrazi* sp. nov., female (IZCAS AR-25147) **A–C** habitus in dorsal, ventral, and lateral views **D–F, H** prosoma in dorsal, ventral, lateral, and anterior views **G** abdomen in ventral view. Abbreviation: ldi = labium deep incision; spr = sclerotized posterior ridge. Scale bars: 0.4 mm.

Figs 7 , 8 , 9 , 14G–I , 15E, F , 16C, D

##### Type material.

***Holotype*** ♂: Myanmar, Kachin State, Putao, Hponkanrazi Wildlife Sanctuary, around Ziradum; 27°34.499'N, 97°03.546'E; elevation ca 1100 m; 19.XII.2016; Wu J. leg. (IZCAS AR-25141). ***Paratypes*** 1♀: Myanmar, same data as for holotype (IZCAS AR-25142); 2♂1♀: roadside between Wasadum and Ziradum; 27°32.305'N, 97°07.537'E; elevation ca 980 m; 12.XII.2016; Wu J. leg. (IZCAS AR-25143-25144-25145); 1♂: same data as preceding; 27°32.767'N, 97°07.283'E; elevation ca 970 m; 12.XII.2016; Wu J. leg. (IZCAS AR-25146); 3♀: around Ziradum Village; 27°33.465'N, 97°06.580'E; 1051 m; 8.V.2017; Wu J. leg. (IZCAS AR-25147-25148-25149); 1♀: same data as preceding; 27°35.305'N, 97°04.893'E; elevation ca 1140 m; 13.V.2017; Wu J. leg. (IZCAS AR-25150).

##### Diagnosis.

The new species is similar to *T. gongshan* Tong, Zhang & Li, 2019 but can be distinguished by the forked dorsal branch of the embolus system (Fig. 8E), the curved, strongly sclerotized posterior ridge (spr) of the female’s epigastric area (Fig. 9G), and the reticulate carapace (Figs 7D, F, 9D, F) of both sexes. *Trilacuna gongshan* has three long, tooth-like lobes in the embolus system, without a curved, strongly sclerotized posterior ridge in the female’s epigastric area, and with a granulate carapace in both sexes (Tong et al. 2019: figs 10–12, 24G, H).

##### Description.

**Male. *Body***: yellow, chelicerae and sternum lighter; habitus as in Figure 7A–C; body length 1.49. ***Carapace***: 0.74 long, 0.60 wide; sides finely reticulate; lateral margin rebordered (Fig. 7C). ***Eyes***: ALE largest; PLE and PME nearly equal in size; ALE–PLE separated by less than ALE radius; PME touching each other; posterior eye row recurved as viewed from above, procurved as viewed from front (Fig. 7D, H). ***Clypeus***: height about 1.25 times of ALE diameter. ***Mouthparts*** (Figs 7E, H, 16C, D). ***Sternum*** (Fig. 7E). ***Abdomen***: 0.63 long, 0.48 wide; booklung covers ovoid, surface smooth (Fig. 7C); dorsal scutum not fused with epigastric scutum; apodemes absent; posterior spiracles not connected by groove; epigastric region with patches between the posterior spiracles (Fig. 7G). ***Palp*** (Figs 8, 14G–I): orange; 0.52 long (0.15, 0.09, 0.13, 0.15); femur greatly swollen (width/length = 0.74) (Fig. 8A, B); bulb oval, stout, tapering apically; embolus system (Fig. 8E, F, H) with a forked dorsal branch (db) and a small ventral lobe (svl) in prolateral view, with a small median branch (mb) and a lateral branch (lb) in retrolateral view.

**Female.** Same as male except as noted. ***Habitus***: as in Figure 9A–C. ***Body***: length 1.43. ***Carapace***: 0.70 long, 0.57 wide. ***Abdomen***: 0.76 long, 0.45 wide. ***Epigastric area*** (Figs 9G, 15E): with a curved, strongly sclerotized posterior ridge (spr). ***Endogyne*** (Fig. 15F): with narrow, transverse sclerite (tsc), an anterior stick-shaped sclerite (as), and a posterior small globular structure (glo); transverse bars (tba) with two lateral apodemes (ap).

##### Etymology.

The specific name is a noun in apposition taken from the type locality.

##### Distribution.

Known only from the type locality.

#### Trilacuna loebli

Taxon classificationAnimaliaAraneaeOonopidae

Grismado & Piacentini, 2014

7F56EAB9-8F44-57DB-AE09-BC5A0C2986D6

Figs 10 , 15G , 15H

Trilacuna loebli Grismado & Piacentini, in Grismado et al. 2014: 44, fig. 35A–I

##### Material examined.

1♀, Myanmar, Kachin State, Putao, roadside between Wasadum and Ziradum; 27°32.305'N, 97°07.537'E; elevation ca 980 m; 12.XII.2016; Wu J. leg. (IZCAS AR-25156).

##### Diagnosis.

Females of this species can be distinguished from other congeners by the semicircular plate of the epigastric area and the worm-shaped globular structure of the endogyne (Fig. 15G, H).

##### Description.

See Grismado et al. 2014.

##### Distribution.

India (Assam); Myanmar.

##### Variation.

**Figure 10.**
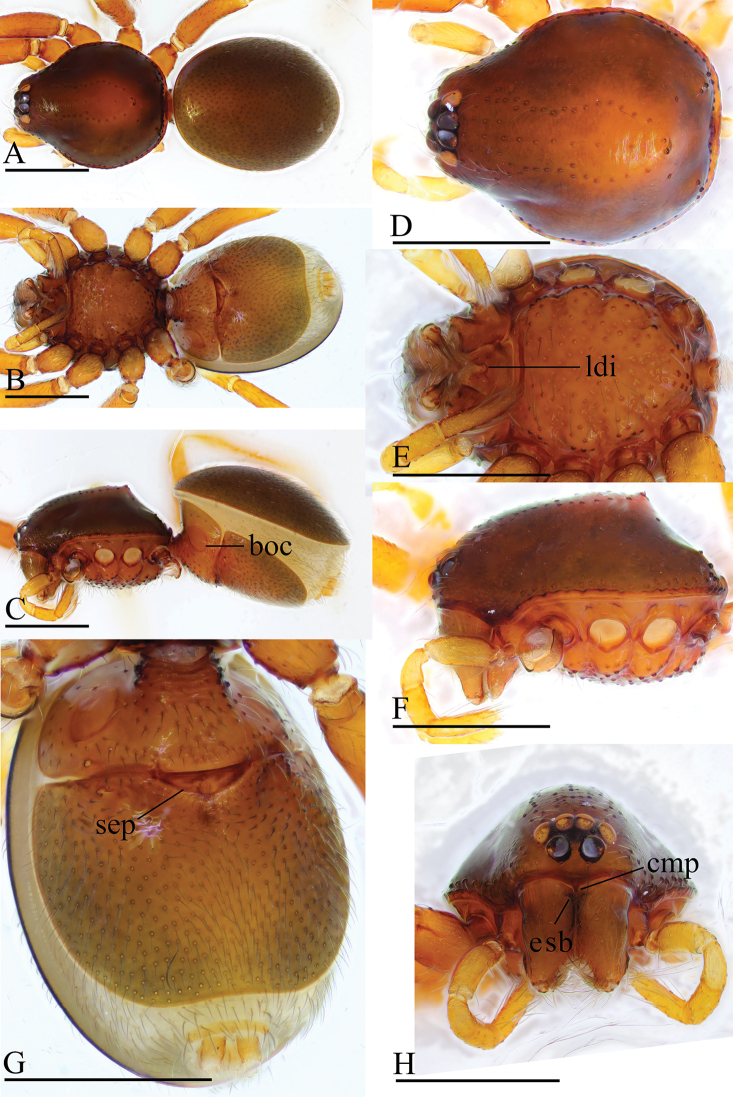
*Trilacuna loebli*, female (IZCAS AR-25156) **A–C** habitus in dorsal, ventral, and lateral views **D–F, H** prosoma in dorsal, ventral, lateral, and anterior views **G** abdomen in ventral view. Abbreviations: boc = booklung covers; cmp = clypeus median projection; esb = elevated seta base; ldi = labium deep incision; sep = semicircular plate. Scale bars: 0.4 mm.

The specimens from Myanmar have a reticulate carapace and a nearly straight posterior eye row in dorsal view (Fig. 10D). By contrast, the specimens from India have a granulate carapace, and the posterior eye row is slightly recurved in dorsal view (Grismado et al. 2014: figs 35H, I).

#### Trilacuna triseta

Taxon classificationAnimaliaAraneaeOonopidae

Tong & Li sp. nov.

928E0B69-FD72-5DC1-A003-7E8A37BF3908

http://zoobank.org/C04150ED-AD13-41F8-A86D-EE9FDD9B02D0

**Figure 11.**
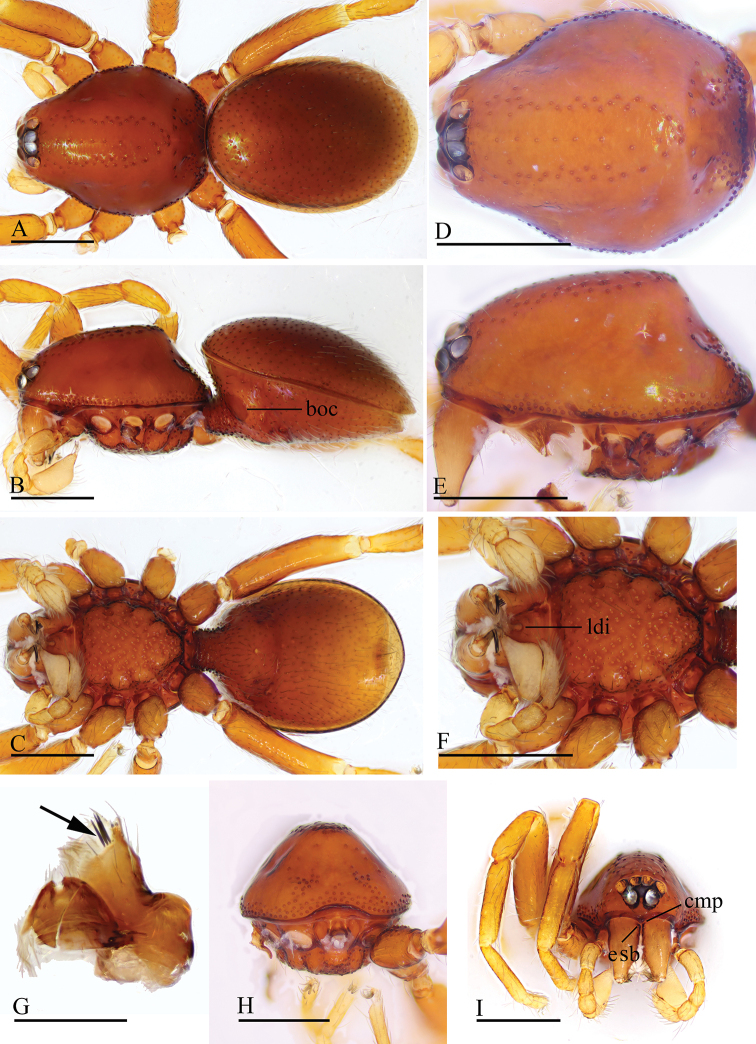
*Trilacuna triseta* sp. nov., male holotype **A–C** habitus in dorsal, lateral, and ventral views **D–F, H, I** prosoma in dorsal, lateral, ventral, posterior, and anterior views **G** labium and endites in ventral view (arrow shows the three black, strong setae). Abbreviations: boc = booklung covers; cmp = clypeus median projection; esb = elevated seta base; ldi = labium deep incision. Scale bars: 0.4 mm (**A–F, H, I**); 0.2 mm (**G**).

**Figure 12.**
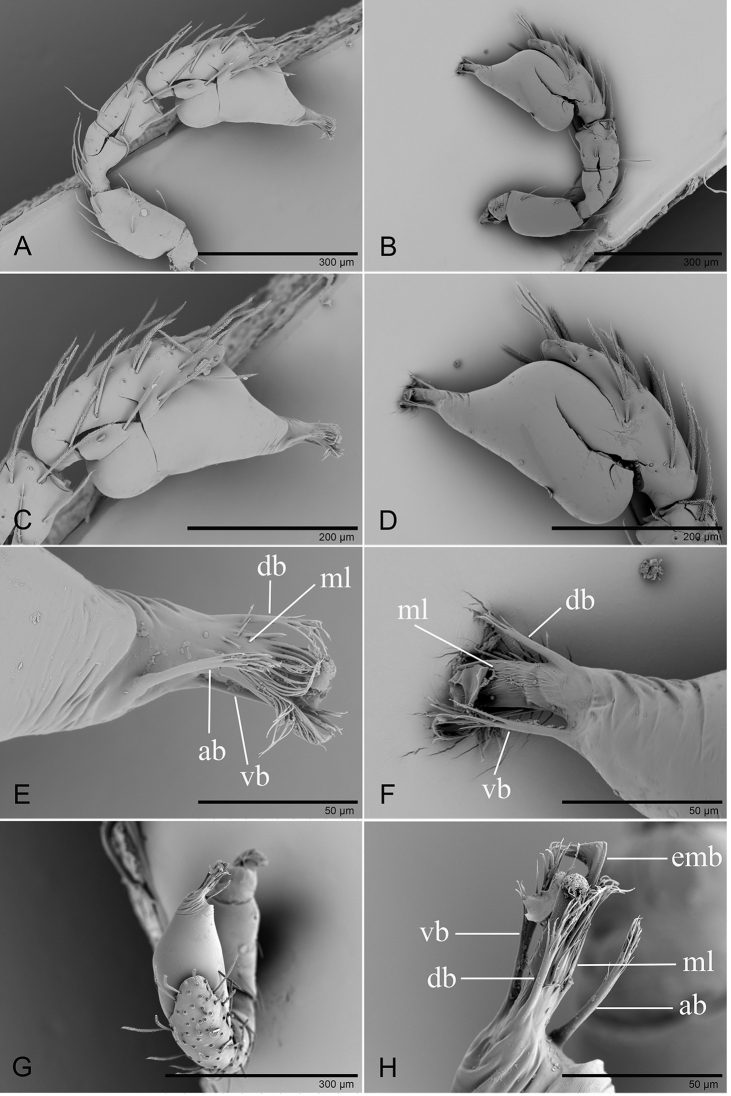
*Trilacuna triseta* sp. nov., male holotype, left palp, SEM**A, B, G** prolateral, retrolateral, and dorsal views **C, D** palpal bulb in prolateral and retrolateral views **E, F, H** distal part of palpal bulb in prolateral, retrolateral, and dorsal views. Abbreviations: ab = anterior branch; db = dorsal branch; emb = embolus; ml = median lobe; vb = ventral branch.

Figs 11 , 12 , 14J–L , 16G , 16H

##### Type material.

***Holotype*** ♂: Myanmar, Kachin State, Putao, Hponkanrazi Wildlife Sanctuary; 27°32.032'N, 97°00.036'E; elevation ca 2010 m; 15.V.2017; Wu J. and Chen Z. leg. (IZCAS AR-25155).

##### Diagnosis.

The new species is similar to *T. bilingua* Eichenberger, 2011 but can be distinguished by the three black, thick setae on the endites of the male (Figs 11G, 16G, 16H) and the slender anterior branch (ab) of the embolus system (Fig. 12E, 12F, 12H). *Trilacuna bilingua* lacks black, thick setae on the endites, and the embolus system has two very short lobes basally (Eichenberger et al. 2011: fig. 5–6).

##### Description.

**Male. *Body***: reddish brown, chelicerae and sternum lighter, legs yellow; habitus as in Figure 11A–C; body length 1.97. ***Carapace***: 0.95 long, 0.74 wide; sides smooth; lateral margin rebordered (Fig. 11B); posterior surface with several large setal bases (Fig. 11H). ***Eyes***: ALE largest; PLE and PME nearly equal in size; ALE–PLE separated by less than ALE radius; PME touching each other; posterior eye row recurved as viewed from above, procurved as viewed from front (Fig. 11D, I). ***Clypeus***: height about 0.85 times of ALE diameter, with a triangular, pointed, median projection (cmp). ***Mouthparts*** (Figs 11F, I, 16G, H): endites with three thick, black setae. ***Sternum*** (Fig. 11F). ***Abdomen***: 1.05 long, 0.80 wide; booklung covers ovoid, surface smooth (Fig. 11B); dorsal scutum not fused to epigastric scutum; apodemes present; posterior spiracles connected by groove (Fig. 11C). ***Palp*** (Figs 12, 14J–L): orange; 0.72 long (0.17, 0.11, 0.11, 0.33); femur elongated (width/length = 0.53) (Fig. 12A, B); bulb oval, tapering apically; embolus system (Fig. 12E, 12F, 12H) with an anterior branch (ab), a dorsal branch (db), a ventral branch (vb), and a median lobe (ml) in prolateral view.

**Female.** Unknown.

##### Etymology.

The species epithet is a noun in apposition composed of the Latin words *tri* (three) and *seta* and refers to the three black, thick setae on the male’s endites.

##### Distribution.

Known only from the type locality.

##### Remarks.

**Figure 13.**
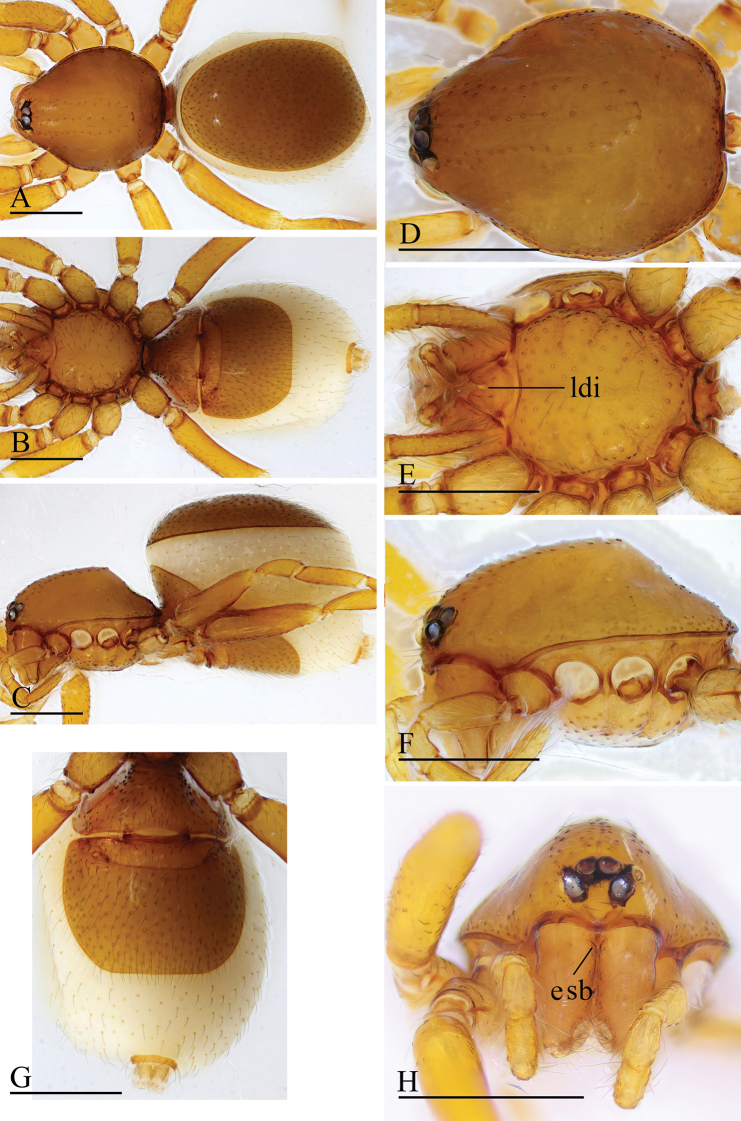
*Trilacuna zhigangi* sp. nov., female holotype **A–C** habitus in dorsal, ventral, and lateral views **D–F, H** prosoma in dorsal, ventral, lateral, and anterior views **G** abdomen in ventral view. Abbreviation: esb = elevated seta base; ldi = labium deep incision. Scale bars: 0.4 mm.

There are two species reported from a single female specimen in this study, and one described from a single male: *T. loebli* Grismado & Piacentini, 2014, *T. zhigangi* Tong & Li, sp. nov., and *T. triseta* Tong & Li, sp. nov., respectively. The following characters indicate that neither of the two females are conspecific with *T. triseta* Tong & Li, sp. nov. *T. loebli* is dark brown (Fig. 10A) with a reticulated carapace (Fig. 10D), and *T. zhigangi* has small eyes (Fig. 13A) and lacks the triangular, pointed, median projection of the clypeus (Fig. 13H). The male, *T. triseta* Tong & Li, sp. nov. has a reddish-brown body, a smooth carapace, normal-sized eyes, and a triangular, pointed, median projection (Fig. 11A, D, I).

#### Trilacuna zhigangi

Taxon classificationAnimaliaAraneaeOonopidae

Tong & Li sp. nov.

5A5C50E8-18E9-500B-8AF5-0E121752AC82

http://zoobank.org/08344EDB-66A5-4D7F-BC2C-330FE3F2619B

Figs 13 , 15I , 15J

##### Type material.

**Holotype** ♀: Myanmar, Kachin State, Putao, Hponkanrazi Wildlife Sanctuary; 27°31.592'N, 96°58.266'E; elevation ca 2470 m; 15.V.2017; Wu J. and Chen Z. leg. (IZCAS AR-25157).

##### Diagnosis.

The new species is similar to *T. bangla* Grismado & Ramírez, 2014 but can be distinguished by the short, lateral apodemes (they do not reach the groove connecting the posterior spiracles) and the stick-shaped anterior sclerite of the endogyne (Fig. 15J). *Trilacuna bangla* has very long lateral apodemes (they distinctly extend beyond the groove connecting the posterior spiracles), and the anterior sclerite has long arms (Grismado et al. 2014: fig. 48A).

##### Description.

**Female. *Body***: yellow, chelicerae and sternum lighter, legs yellow; habitus as in Figure 13A–C; body length 2.02. ***Carapace***: 0.86 long, 0.71 wide; sides reticulate; lateral margin rebordered (Fig. 13F). ***Eyes***: ALE largest; PLE and PME nearly equal in size; ALE–PLE separated by less than ALE radius; PME touching each other; posterior eye row recurved as viewed from above, procurved as viewed from front (Fig. 13D, H). ***Clypeus***: height about 1.2 times of ALE diameter. ***Mouthparts*** (Figs 13E, H). ***Sternum*** (Fig. 13E). ***Abdomen***: 1.20 long, 0.87 wide; booklung covers ovoid, surface smooth; postgastric scutum short, covering about 2/3 abdomen length. ***Epigastric area*** (Figs 13G, 15I): surface without external features. ***Endogyne*** (Fig. 15J): with narrow, transverse sclerite (tsc), an anterior stick-shaped sclerite (as), and a posterior small globular structure (glo); transverse bars (tba) with two lateral apodemes (ap).

**Male.** Unknown.

##### Etymology.

The species is named after Mr Zhigang Chen, one of the collectors of the holotype.

##### Distribution.

Known only from the type locality.

**Figure 14.**
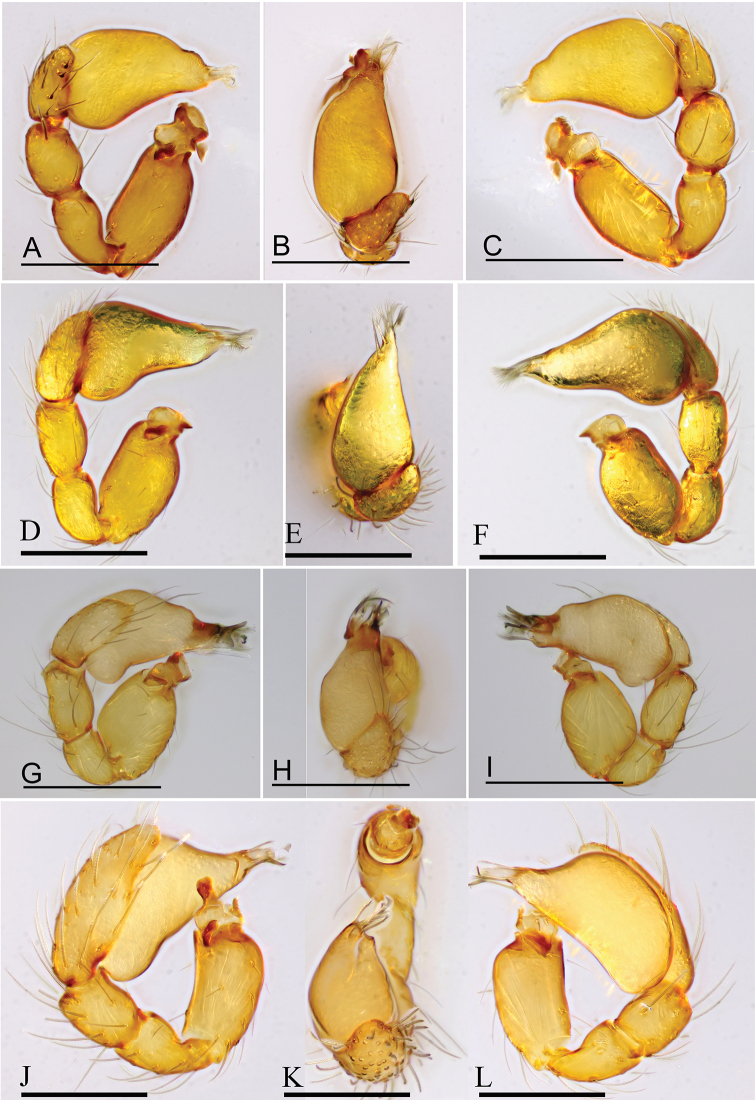
*Trilacuna* spp., left male palp **A–C***T. besucheti***D–F***T. changzi* sp. nov. **G–I***T. hponkanrazi* sp. nov. **J–L***T. triseta* sp. nov. **A, D, G, J** prolateral views **B, E, H, K** dorsal views **C, F, I, L** retrolateral views. Scales: 0.2 mm.

**Figure 15.**
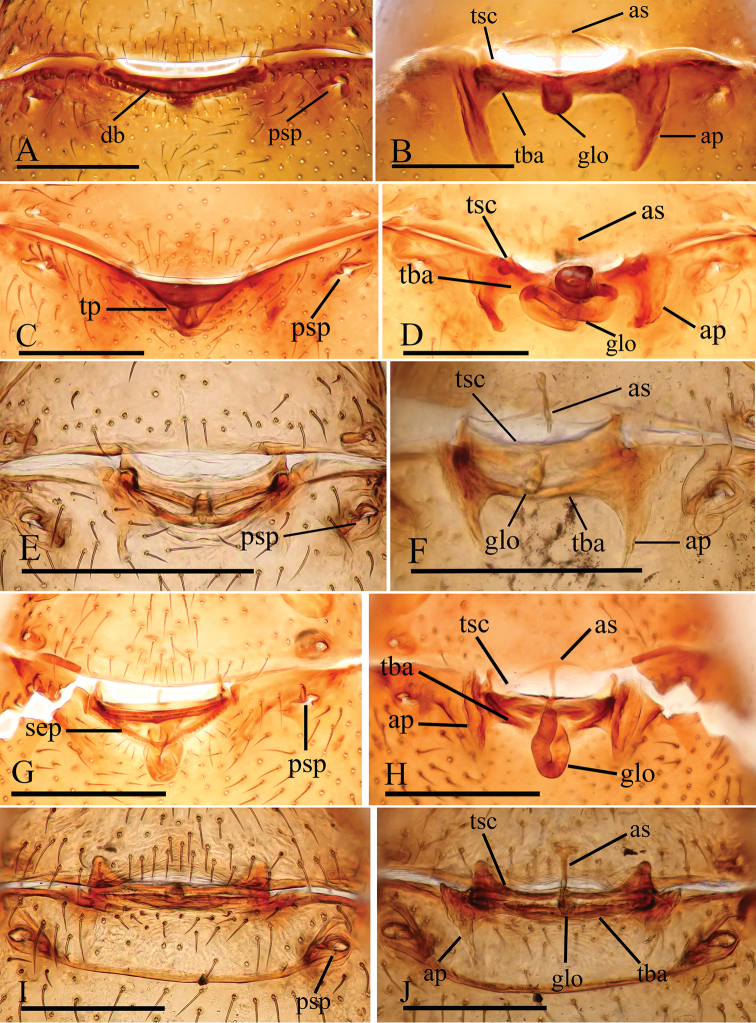
*Trilacuna* spp., female copulatory organ **A, B***T. besucheti***C, D***T. changzi* sp. nov. **E, F***T. hponkanrazi* sp. nov. **G, H***T. loebli***I, J***T. zhigangi* sp. nov. **A, C, E, G, I** ventral view **B, D, F, H, J** dorsal view. Abbreviations: ap = apodeme; as = anterior sclerite; db = darkened band; glo = globular structure; psp = posterior spiracle; sep = semicircular plate; tba = transverse bars; tp = triangular plate; tsc = transverse sclerite. Scales: 0.2 mm.

**Figure 16.**
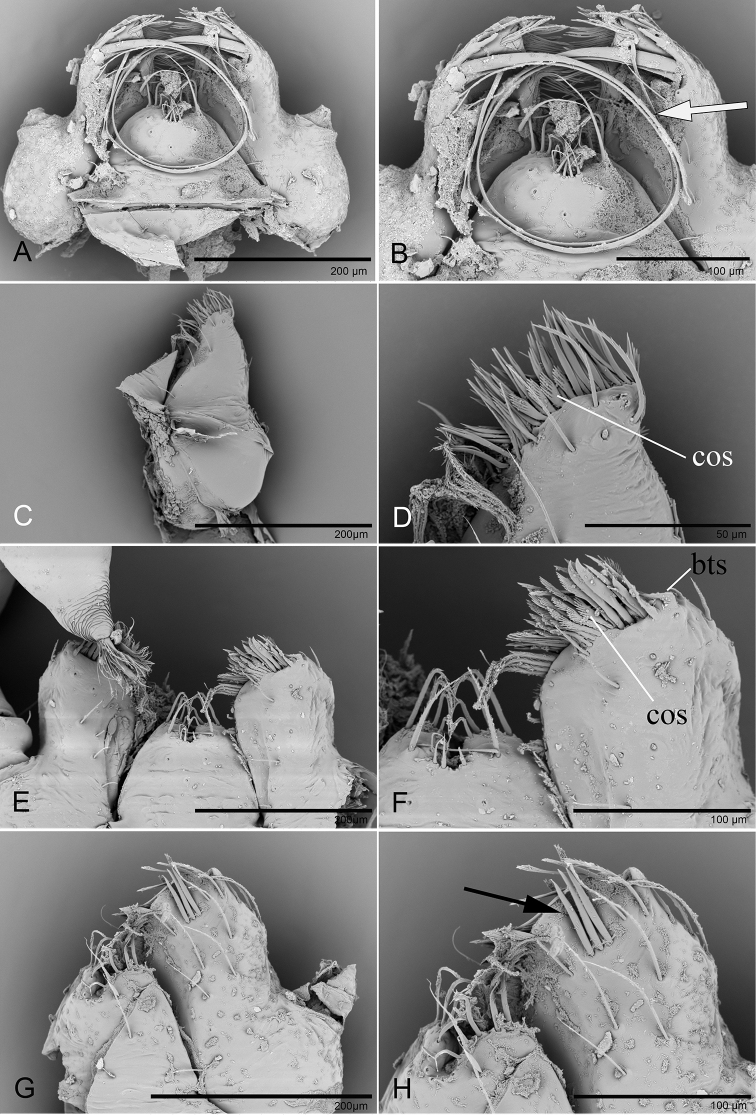
*Trilacuna* spp., male labium and endites, ventral view **A, B***T. changzi* sp. nov., white arrow shows the long, strongly curved spines **C, D***T. hponkanrazi* sp. nov. **E, F***T. besucheti***G, H***T. triseta* sp. nov., black arrow shows the three black, strong setae. Abbreviations: bts = bent thick setae; cos = comb-like setae.

## Supplementary Material

XML Treatment for Trilacuna

XML Treatment for Trilacuna besucheti

XML Treatment for Trilacuna changzi

XML Treatment for Trilacuna hponkanrazi

XML Treatment for Trilacuna loebli

XML Treatment for Trilacuna triseta

XML Treatment for Trilacuna zhigangi
